# Water availability shapes temporal patterns of extrafloral nectar secretion and ant visitation to a Neotropical legume

**DOI:** 10.1111/plb.70164

**Published:** 2026-01-05

**Authors:** B. Melati, C. Souza, A. Nogueira, L. C. Leal

**Affiliations:** ^1^ Centro de Ciências Naturais e Humanas Universidade Federal do ABC Santo André Brazil; ^2^ Departamento de Ecologia e Biologia Evolutiva Universidade Federal de São Paulo Diadema São Paulo Brazil; ^3^ Laboratório de Interações Planta‐Animal (LIPA) Centro de Ciências Naturais e Humanas, Universidade Federal do ABC São Bernardo do Campo Brazil

**Keywords:** Ant–plant interaction, herbivory, mutualism, plant defence, plant water potential, water deficit

## Abstract

- Mutualistic plants use non‐structural sugar (NSC) to produce carbon‐based resources to reward partners. Then, any factor compromising NSC plant supplies should increase the relative costs of these rewards for plants. Drought, for instance, can initially reduce plant growth but not necessarily photosynthesis, which could boost NSC supplies. However, if drought persists and compromises plant water status, photosynthetic rates decline, reducing NSC reserves and potentially impacting the plant's investment in mutualistic rewards. We hypothesized that plants would initially increase extrafloral nectar investment – a sugary solution attractive to ants – at the onset of water shortage, increasing their attractiveness to ants. However, such investment and attractiveness gradually decrease over time as drought lasts and the plant water status is compromised.
- Here, we experimentally manipulated soil water availability and water potential of *Chamaecrista nictitans* (Fabaceae) and evaluated the effects of soil water decline over time on patterns of extrafloral nectar secretion and plant visitation by ants.
- We observed that *C. nictitans* had more active extrafloral nectaries producing more concentrated nectar at drought onset, while plant water potential was not affected. However, it gradually declined as the water shortage progressed, and plant water potential declined. Ant visitation followed a similar temporal pattern, peaking at the experiment onset and diminishing over time as the drought lasted.
- These findings emphasize the pivotal role of drought severity in shaping the temporal dynamics of extrafloral nectar secretion and likely other carbohydrate‐based mutualistic rewards, shedding light on the physiological mechanisms regulating mutualisms.

## Graphical abstract

Drought duration affects ant–plant interactions: extrafloral nectar quality initially increases and subsequently declines, and ant interaction patterns closely track these nectar dynamics.

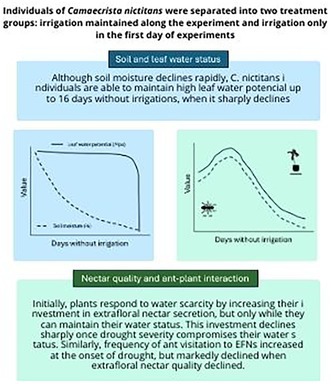

## INTRODUCTION

Mutualistic interactions can be interpreted as biological markets in which individuals of different species offer to their partners' services or rewards (commodities) that are relatively cheap to produce, in exchange for commodities that are expensive or impossible to obtain otherwise (Schwartz & Hoeksema 1998; Hammerstein & Noë 2016). This market perspective highlights two paramount mechanisms regulating the eco‐evolutionary dynamic of mutualistic interactions: (1) mutualistic partners need to afford the costs of producing and offering mutualistic commodities (Bronstein 2001), and (2) the positive outcome of the interaction depends on the extent to which benefits outweigh such costs for each partner (Keeler 1985). The costs and benefits associated with mutualistic interactions are context‐dependent (Bronstein 1994), resulting in considerable variability in the outcomes of such interactions over time and space (Chamberlain *et al*. 2014; Nogueira *et al*. 2015, 2016; Leal & Peixoto 2017). Therefore, understanding the ecological factors driving the costs and benefits of mutualistic commodities to partners is a fundamental task to predict the outcome of different types of mutualisms (Bronstein 2009; Hammerstein & Noë 2016), especially in the current scenario of global climatic changes.

Plants commonly offer carbohydrate‐based rewards to their mutualistic partners, and the production of these rewards relies on the supply of non‐structural carbohydrates (NSCs), including starch, soluble sugar and fructans (Pringle 2015). These carbohydrates are assimilated via photosynthesis and are used to produce mutualistic rewards and maintain other plant functions such as growth, respiration, storage, and stress tolerance (Pringle 2015). Any factor affecting the NSC assimilation and storage, or the opportunity cost of allocating NSC to mutualists instead of other plant functions, is expected to indirectly affect mutualistic costs and, ultimately, the outcome of the interaction for plants (Pringle 2015). Soil water availability is an excellent example of these factors as its decline can directly affect plant photosynthetic rates, progressively increasing the imbalance between NSC supply from photosynthesis and its utilization for sustaining plant functions (Chaves *et al*. 2002; McDowell 2011). Interestingly, plants also use NSC to mitigate the metabolic consequences of reduced carbon input resulting from decreased photosynthesis during drought conditions (Santos *et al*. 2021; Signori‐Müller *et al*. 2021). Consequently, the negative impact of drought on plant photosynthetic rates is expected to increase carbon allocation trade‐off as drought lasts, leading plants towards carbon starvation (*i.e*., failure in maintaining metabolism due to prolonged negative carbohydrate balance, sensu McDowell 2011). In such a scenario, changes in NSC supply and allocation during drought periods can profoundly impact the costs of mutualistic rewards in plants and, ultimately, the plant's capacity to invest in and derive benefits from these interactions.

Over time, the decline in water availability has a non‐linear effect on plant photosynthetic rates and NSC reserves. Although plant responses to drought can vary among species, it is generally expected that drought causes greater reductions in growth than photosynthesis and greater reductions in photosynthesis than maintenance respiration (McDowell 2011) at the beginning of water stress, leading to an initial NSC surplus (Chapin *et al*. 1990; Körner 2003; McDowell 2011). If the drought persists, the photosynthesis finally decreases, depleting the NSC reserves and impacting other plant functions. Considering that mutualistic rewards rely on NSC availability in plant tissues (Pringle 2015), it is possible that the plant's investment in mutualistic rewards and consequently its production will exhibit a similar non‐linear pattern. The initial surplus of NSC can increase the mutualistic reward, favouring mutualistic interactions. However, if the drought lasts, mutualistic rewards can become increasingly costly, leading to a progressive decline of plant investment in rewards up to its complete cessation. As plant investment in mutualistic rewards is commonly related to the magnitude of benefits obtained from the interaction (Wyatt *et al*. 2014), drought impact on plant's carbon dynamics can be a major mechanism regulating the observed changes in plant interactions with mutualistic partners and the outcomes of these interactions along temporal and spatial water gradients (Contreras *et al*. 2013; Leal & Peixoto 2017; Smith *et al*. 2018; Bascompte *et al*. 2020; Câmara *et al*. 2022). Despite that, empirical evidence connecting plant physiological responses to drought and the dynamic of mutualistic interactions in nature remains scarce (but see Ávila‐Argáez *et al*. 2019). As climate change is predicted to increase the frequency and severity of drought (Chiang *et al*. 2021), this knowledge gap strongly compromises our ability to predict the future of plant populations relying on ecological services provided by mutualistic partners.

In this study, we investigated the effects of the declining soil water availability to plants over time on the plant investment in the secretion of extrafloral nectar – a sugary aqueous solution secreted by extrafloral nectaries (EFNs). This nectar rewards predatory arthropods, mainly ants, that can defend plants against herbivores (Heil 2015). Over 4,000 plant species worldwide bear EFNs (Weber & Keeler 2013). These species occur in a broad range of habitats, being relatively common in seasonal dry environments (*e.g*., Aranda‐Rickert *et al*. 2014; Leal *et al*. 2015, 2017; Luo *et al.* 2023). The cost of extrafloral nectar secretion has been considered low or even negligible for plants with EFNs (O'Dowd 1979; Bronstein 2001). However, like any other carbon‐based mutualistic reward, its relative cost is likely determined by the plant carbon supply, expressly NSC reserves (Pringle 2015; Xu & Chen 2015). Therefore, we hypothesized that temporal patterns of extrafloral nectar secretion during drought reflect the effect of water availability decline on plant physiological conditions. Specifically, we predicted an increase in extrafloral nectar secretion due to the NSC surplus at the drought onset, followed by a decrease as drought persists. Since the effectiveness of this anti‐herbivory defence relies on ant responses to extrafloral nectar and that these responses are driven by extrafloral nectar features (*i.e*., volume and sugar concentration) (Blüthgen *et al*. 2004; Blüthgen & Fiedler 2004; Lange *et al*. 2013), we also hypothesized that variation in patterns of extrafloral nectar secretion along drought determines patterns of ant attendance on EFN‐bearing plants. Therefore, ant attendance is expected to be higher at drought onset, decreasing as the drought persists (Heil 2015).

## METHODS

### Model plant species

To investigate the impact of soil water scarcity on extrafloral nectar secretion, we used the self‐compatible annual ruderal shrub *Chamaecrista nictitans* (L.) Moench (Fabaceae‐Caesalpinioideae) as a model species (Fig. S1a) (Gottsberger & Silberbauer‐Gottsberger 1988; Lee 1989). These plants bear vascularized EFNs on the adaxial surface of leaf petioles (Gleason & Cronquist 1963) (Fig. S1b), attracting protective ants as a defence against herbivores (Fig. S1c). The EFNs' vascularization likely provides a dedicated supply of photoassimilates to EFNs (Coutinho *et al*. 2022), which can facilitate secretion even in drought conditions (*e.g*., Ávila‐Argáez *et al*. 2019). The secretion by *C. nictitans* EFNs can be induced by herbivory, demonstrating their adaptability to external factors (Chinarelli *et al*. 2022), which makes *C. nictitans* a suitable model for our study.

### Plant cultivation and experimental design

In our experiments, we used plants grown from seeds of 10 distinct mother plants from an experimental *C. nictitans* population maintained by our research group at Universidade Federal do ABC (UFABC), São Bernardo do Campo, SP, Brazil (23°40′ S, 46°33′ W). This experimental population is composed of plants transplanted from a natural *C. nictitans* population from Parque Estadual do Juquery – a protected area of Cerrado vegetation in São Paulo state Brazil. Plants from the experimental population had not been submitted to any previous experimental treatments and had been cultivated only as source of seeds. The summer (December–March) is the wettest period of the year in our study site, when the average temperature and precipitation are, on average, 24 °C (±1.5 °C, SD) and 190 mm month^−1^ (±70 mm), respectively (INMET 2023). Winter (June–September) is the driest season, when the mean temperature and precipitation are 16 °C (±2.5 °C) and 40 mm month^−1^ (±20 mm), respectively (INMET 2023). Within our experimental population, seed germination and seedling emergence of *C. nictitans* occur at the start of the wet season (October–December). These plants exhibit rapid growth and a short non‐reproductive juvenile phase (~4–5 months). Once flowering begins, they produce flowers and fruits continuously until they die, during the mid‐dry season (August–September). Seeds produced during each reproductive cycle are incorporated into the soil seed bank, where they survive until the next wet season, restarting the *C. nictitans* annual cycle.

In December 2019, when seedlings started to emerge in our experimental population, we initiated the cultivation of our experimental *C. nictitans* plants at the Universidade Federal do ABC (UFABC) greenhouse. We scarified the harvested seeds using sandpaper and planted three seeds from each mother plant in four different 8 l pots, totalling 40 experimental pots (4 pots × 10 mother plants). We filled the pots with a mixture of 70% sand and 30% garden soil enriched with organic matter and macronutrients (Terra Vegetal ABC GARDEN) (see Chinarelli *et al*. 2022). In addition to the initial 40 pots, we planted a second set of seeds from four other mother plants in eight spare pots. We used these four additional pots to track variations in soil water content and plant water status along the experiment simulating the temporal decline of water availability to plants (Fig. S2) (see Plant water status section for more details). After 2 months, we selectively retained the juvenile plants with most expanded leaves, removing the other two plants from all 48 pots. Then, we got a final set of 48 juvenile plants, one per pot, from which eight were exclusively used to track soil water content and plant water status along our experiment (Fig. S2) (see Plant water status section). During the plant growth phase, we watered the plants daily using a drip mechanism until the start of the experiment. While in the greenhouse, the plants were maintained between 22 and 28 °C, with relative air humidity of 70% (Fig. S2).

After 6 months of seed germination (July 2020 – dry season), all plants had begun their reproductive period, with flower production underway. By this time, we relocated all 48 pots containing a single seedling from the greenhouse to an experimental field site within the UFABC campus (Fig. S2). In this area, the plants faced an average temperature of 18.45 °C (+1.71 °C), average relative air humidity of 79.18% during the day and 93.75% during the night, and average daily precipitation of 1.10 mm (+2.09 mm) along the 21 days of experiment. The temperature, humidity and precipitation data in the period of our experiments were extracted from Giovanni (NASA, GES DISC), respectively from: GLDAS_CLSM025_DA1_D (Li *et al*. 2019; Li & Rodell 2020), AIRS3STM (AIRS project 2019) and M2T1NXFLX (Pawson 2015). At this point, the plants had, on average, 15.30 ± 3.31 leaves. The site is a human‐modified habitat dominated by shrubs and herbaceous plants, where interactions between ant species and EFNs‐bearing plant species, including *C. nictitans* (Chinarelli *et al*. 2022), are frequent. In the field, we placed the first set of plants (40 plants) 10 m apart, as a standardized distance used in experiments assessing ant attendance patterns to plant resources (Agosti & Alonso 2000). We placed the plants in open areas, ensuring that all plants from both treatments were exposed to full‐light conditions and far from the influence of the canopy of neighbour plants. This ensured that plants from both treatments remained in similar and nearby areas, reducing any potential differences in herbivory pressure between them. Therefore, variation in shading effects and herbivory may not be a concern between treatments at experiment. The second set of spare plants (eight individuals) was maintained at the same field conditions but clustered and physically separated from the first set.

Still in July 2020, we randomly distributed plants from the same mother plant into two treatment groups: (1) non‐irrigated treatment, in which we submitted the plants to progressive decline of soil water availability (first set = 20 plants, second set = 4 plants; Fig. S2); and (2) irrigated treatment, in which plants had a constant water supply over time (first set = 20 plants, second set = 4 plants; Fig. S2). We accidentally lost one pot after the first day of the survey, resulting in a final count of 39 plants (15 irrigated, 14 non‐irrigated). At this point, plants assigned both treatments, non‐irrigated and irrigated, were in similar conditions and size: 15.3 ± 3.3 and 15.4 ± 3.47 leaves, respectively. We rinsed and watered all plants in the field 1 day after placing them in the experimental area. After that, we watered the plants from the irrigated treatment at 48‐h intervals and suspended the irrigation of plants from non‐irrigated, subjecting them to a progressive drought regime throughout the experiment.

According to the soil retention curve generated by Laboratory of Soil Physics, from Agronomic Institute of Campinas, UNICAMP, our soil reached saturation at a water content representing 45.8% of the total soil dry mass (see soil water retention values in Table S1). To keep irrigated plants well‐watered below the saturation point, we kept the soil moisture for plants under irrigated treatment at 1 kPa = 30.9%, equivalent to soil's field capacity when excess water is drained (Israelsen & West 1922). Throughout the experiment, we monitored soil moisture in both irrigated and non‐irrigated treatments using soil sensors (HH2 model, Delta‐T devices ©). We adjusted the water content only in the pots from irrigated treatment to ensure the maintenance of soil's field capacity along the experiment.

To ensure the soil in non‐irrigated treatment was unaffected by the rain, we placed a plastic lid above and beneath each pot but exposed the plants to the environment (Fig. S1d). We placed the lids on rainy days and removed them on rain‐free days to prevent excessive temperature buildup inside the pots. As rain leads to sampling artefacts due to water soiling the nectar sample and ant foraging, we conducted sampling only after two consecutive days without rainfall.

### Plant water status

To evaluate the extent in which our non‐irrigation treatment influenced the water supply for our plants and *C. nictitans* water content, we run an experiment in which we monitored the soil water content and water potential of irrigated and non‐irrigated plants. For this experiment, we monitored the leaf water status from of the eight spare plants from the second set of pots (irrigated: n = 4; non‐irrigated: n = 4) in six different days over the 21 days of the experiment, always at 1:00 pm. This monitoring started in July 2020 and was conducted simultaneously with the experiment evaluating the effect of irrigation suspension on EFNs activity. This experiment occurred simultaneously to those for evaluation of drought effect on extrafloral nectar secretion and ant visitation. Therefore, plants from irrigated and non‐irrigated treatment used to monitored plant water status were maintained under the same conditions of irrigated and non‐irrigated plants of the main experiment, described in the subtopic Plant cultivation and experimental design. We collected one leaf from each plant at the base of the petiole, consistently collecting leaves from the same stem node at each sampling in each group. To determine the leaf water potential, we placed individual leaves in a Scholander *et al*. (1965) pressure chamber to measure the pressure required to force water out of the cut region of petiole. The pressure is measured in megapascals (MPa). The lower and more negative MPa values, the higher the pressure required to expel water from the leaf, and consequently, the lower the leaf water potential. Given that our Scholander *et al*. (1965) chamber had a maximum pressure capacity of −10 MPa, we determined that leaves were completely dry if no water was expelled up to −7 MPa.

Along the experiments, we measured the soil moisture in the pots of the second set (4 irrigated, 4 non‐irrigated). This procedure allowed us to trace a parallel between the gradual decline of water in the plant's root zone (soil moisture) and the leaf water potential associated with the secretory responses of EFNs along the experiment.

### Extrafloral nectar traits and patterns of ant attendance

We used 16 plants from the first pot set to assess the effects of declining water availability on the temporal pattern of extrafloral nectar secretion by *C. nictitans*. In pilot experiments, we noted a reduction in soil moisture 4 days after the suspension of irrigation. Therefore, we started sampling extrafloral nectar on the fifth day after the irrigation suspension.

We sampled in three different moments along the experiment, with a mean gap of 6 days between them. On each sampling, we counted all EFNs on each plant, classifying them as active whenever we observed traces of extrafloral nectar on its surface (*e.g*., nectar accumulation, shiny surface, recent crystallized sugar) and inactive when dull‐looking. Then, we estimated the proportion of active EFNs per plant. After counting active EFNs, we entirely bagged the same 16 plants overnight (06:00 pm–06:00 am) to prevent any potential bag‐related effects on photosynthesis during the day (Chinarelli *et al*. 2022) and avoid nectar consumption by insects at night (for more details see Chinarelli *et al*. 2022). After bag removal, we collected the accumulated extrafloral nectar from all EFNs of each plant using 1 μl microcapillary (Blaubrand^®^, Wertheim ‐ Germany, disposable). Then, we estimated the volume of extrafloral nectar secreted by the plants. All four plants from one mother plant secreted less than 1 μl of extrafloral nectar in all samplings. Due to this low volume, we excluded them from our analysis and evaluated the features of extrafloral nectar secreted by 12 plants (six from each treatment) from the remaining three mother plants.

To measure the extrafloral nectar concentration (°Brix), we put the entire volume of nectar collected with microcapillaries per plant into a portable Eclipse refractometer for low‐volume samples (Bellingham‐Stanley, UK). We also calculated the total sugar mass (mg) per plant as a proxy of the plant's sugar allocation to extrafloral nectar secretion (Chinarelli *et al*. 2022). Because the refractometer's minimum detection volume is 0.15 μl (Chinarelli *et al*. 2022), we disregard samples lower than 0.15 μl in volume when estimating extrafloral nectar concentration and sugar mass (n = 32; 66%). Most of these excluded samples were collected on the first sampling day, which occurred 4 days after the beginning of the experiment, when only one plant from each treatment secreted more than 0.15 μl. Hence, for the analysis of extrafloral nectar concentration and sugar mass, we considered only the second and third nectar samples, taken on the 11th and 17th days of the experiment, respectively.

To investigate if water availability indirectly affects patterns of ant attendance to *C. nictitans*, we surveyed ant visits in all 40 individual plants in the field, on three non‐consecutive days, approximately 6 days apart. We always performed these surveys 1 day before the extrafloral nectar sampling described above to reduce any potential bias from plant bagging on ant attendance. In each survey, we observed each plant twice during the day, in the early morning (06:00 am) and afternoon (02:00 pm). Each observation lasted for 3 min, with an extension of 2 min if we observed ants feeding on EFNs, resulting in a total observation period of 5 min per sampling. In each survey, we recorded the presence or absence of ant workers from any species and the number of workers from each morphotype on the plants as a proxy of plant attractiveness to ants (see Dussutour & Simpson 2008; Pacelhe *et al*. 2019). Additionally, considering that plants invest more in the secretion of extrafloral nectar in the youngest leaves (see Cronin & Hay 1996), we counted the number of ants patrolling the three most apical pairs of leaves in each plant during 2 min. This variable provides a measure of ant activity on the youngest leaves, considering the frequency of ant circulation around these active EFNs within a standardized timeframe.

### Statistical analysis

#### Evaluating soil water availability and plant water status

As the relationship between soil moisture and water potential over time is non‐linear and can fit different curves, we employed a model selection approach among polynomials of various orders to obtain the curve that best described the soil moisture and water potential data. In these models, we used soil moisture (%), and the leaf's water potential (MPA) separately as our response variables and the interaction between the water availability treatments (irrigated and non‐irrigated) and the sampling day (time) in relation to the onset of irrigation suspension as a continuous predictor factor. Then, we selected the best fitted model to our data using Akaike's Information Criterium (AIC).

#### Effects of water availability on extrafloral nectar traits and ant attendance to plants

To investigate the impact of declining soil water availability over time on extrafloral nectar secretion and ant attendance to plants, we fitted generalized linear mixed models (GLMMs), using the most appropriate error distribution for the data evaluated in each model. In all models, we used the water treatments, sampling day and their interaction as categorical predictor factors. To address the temporal dependence of our sampling, we included plant identity as a random variable in all models.

To evaluate the effects of water availability on extrafloral nectar traits, we built four models using the number of active EFNs per plant, the volume, sugar concentration and sugar mass of the extrafloral nectar secreted by the plants as response variables. To investigate how water availability affected ant attendance to plants, we fitted three models using the presence/absence of workers on plants as a binary response variable and the number of ant workers on plants (ant recruitment) and the number of ants patrolling the most apical leaves (ant activity) as continuous response variables. As ant species markedly differ in the number of workers recruited to food resources (Parr & Gibb 2010; Cerdá *et al*. 2013), the plant attendance by different ant morphotypes could largely bias our results regarding the ant number and activity on the plants. For this reason, we controlled this potential bias by including ant morphotype as a random variable in the models assessing ant number and activity of ants on the plants. For more in‐depth details on the choice of models, see the Supporting Information S1.

## RESULTS

### Effects of soil water availability and plant water status

**Fig. 1:**
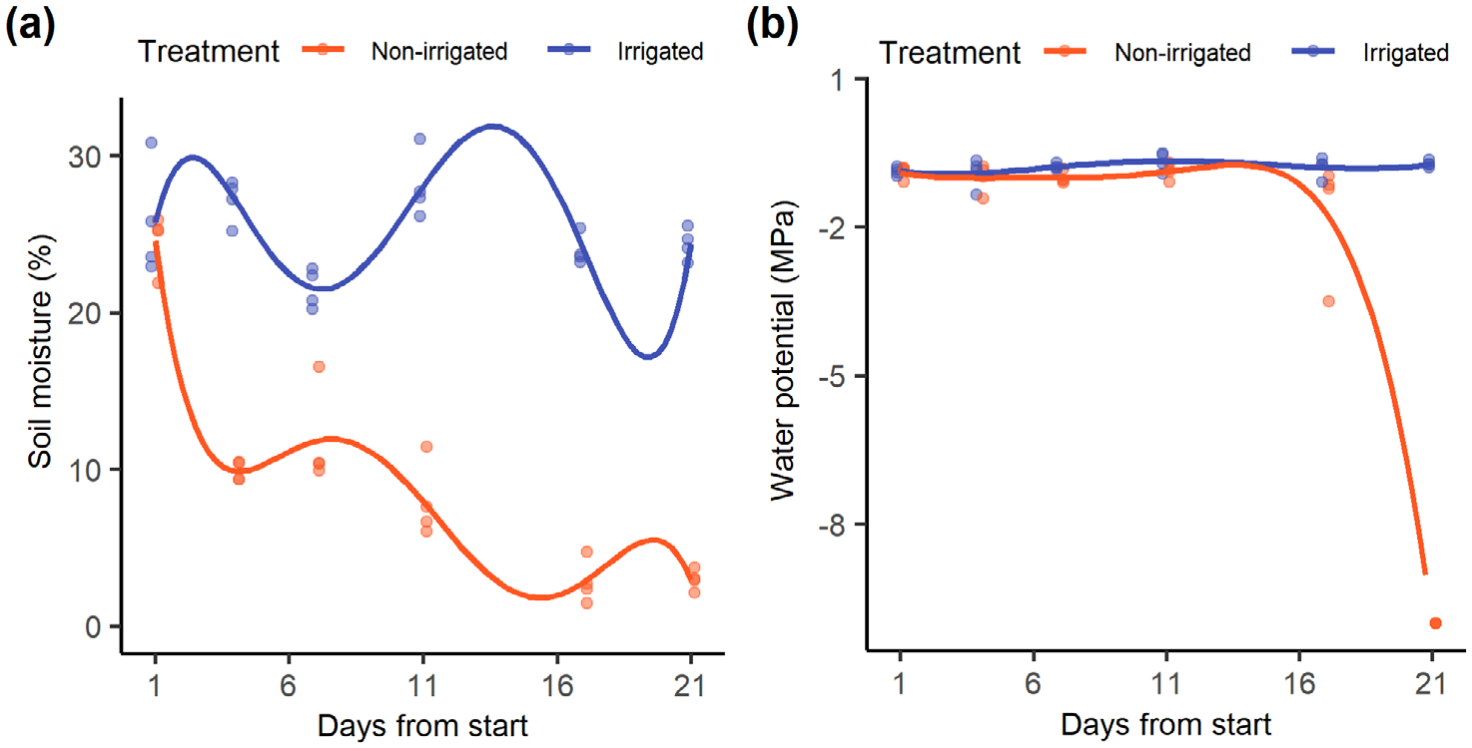
The (a) soil moisture (%) and (b) water potential (MPA) of *Chamaecrista nictitans* (Fabaceae) along 21 days under irrigated (blue) and non‐irrigated (orange) treatments. Soil moisture started to decline on the 4th day after irrigation suspension, while the plant's water potential declined after the 16th day of treatment.

On average, the soil moisture was higher in irrigated (22.50 ± 6.75%; mean ± SD) than non‐irrigated treatment (17.55 ± 9.54%). The temporal patterns of soil moisture differed between the two treatments and were best described by the fifth‐order polynomial model (Table S2). We observed three peaks of soil moisture in the irrigated treatment (approximately at 3, 13 and 21 days of experiment), all followed by a gradual decline. Despite this variation, soil moisture remained relatively constant in the irrigated treatment throughout the experiment, varying between 20.02% and 31.03%. In contrast, soil moisture in the non‐irrigated treatment exhibited a progressive non‐linear decline. Such decline was particularly faster in the first 5 days of the experiment, leading to a soil water content lower than 10% in the fifth day, followed by a slower but progressive decrease, reaching below 4% by the 21st day of the experiment (Fig. 1a). Altogether, this indicates that, as expected, water availability to non‐irrigated plants progressively declined along the experiment, while it remained relatively constant for those in the irrigated treatment.

The mean water potential of plants from irrigated treatment was 1.5 times higher than those from non‐irrigated treatment (−1.12 ± 1.68 MPA and −1.69 ± 2.56 MPA, respectively). The fourth‐order polynomial model provided the best fit for describing the variation in the plant's water potential for both treatments along our experiment (Table S2). In the irrigated treatment, the water potential of the plants remained relatively constant throughout the experiment. However, in the non‐irrigated treatment, there was a drastic decline in the water potential of the plants after the 16th day of irrigation suspension. By this time, the plants showed no wilt signals, but wilt signs started to appear after the 17th day of the experiment. By the 21st day of the experiment, the mean water potential of the non‐irrigated plants was lower than −8, which was 10 times lower than that of irrigated plants. At this point, the plants from the non‐irrigated treatment exhibited completely dry and wrinkled leaves, brown coloured with scorched edges. This decrease indicates that changes in plant water condition were not immediately triggered by the decline in water soil content, being observed 16 days after the progressive reduction in soil moisture (Fig. 1b).

### Effects of water availability on extrafloral nectar traits

**Fig. 2:**
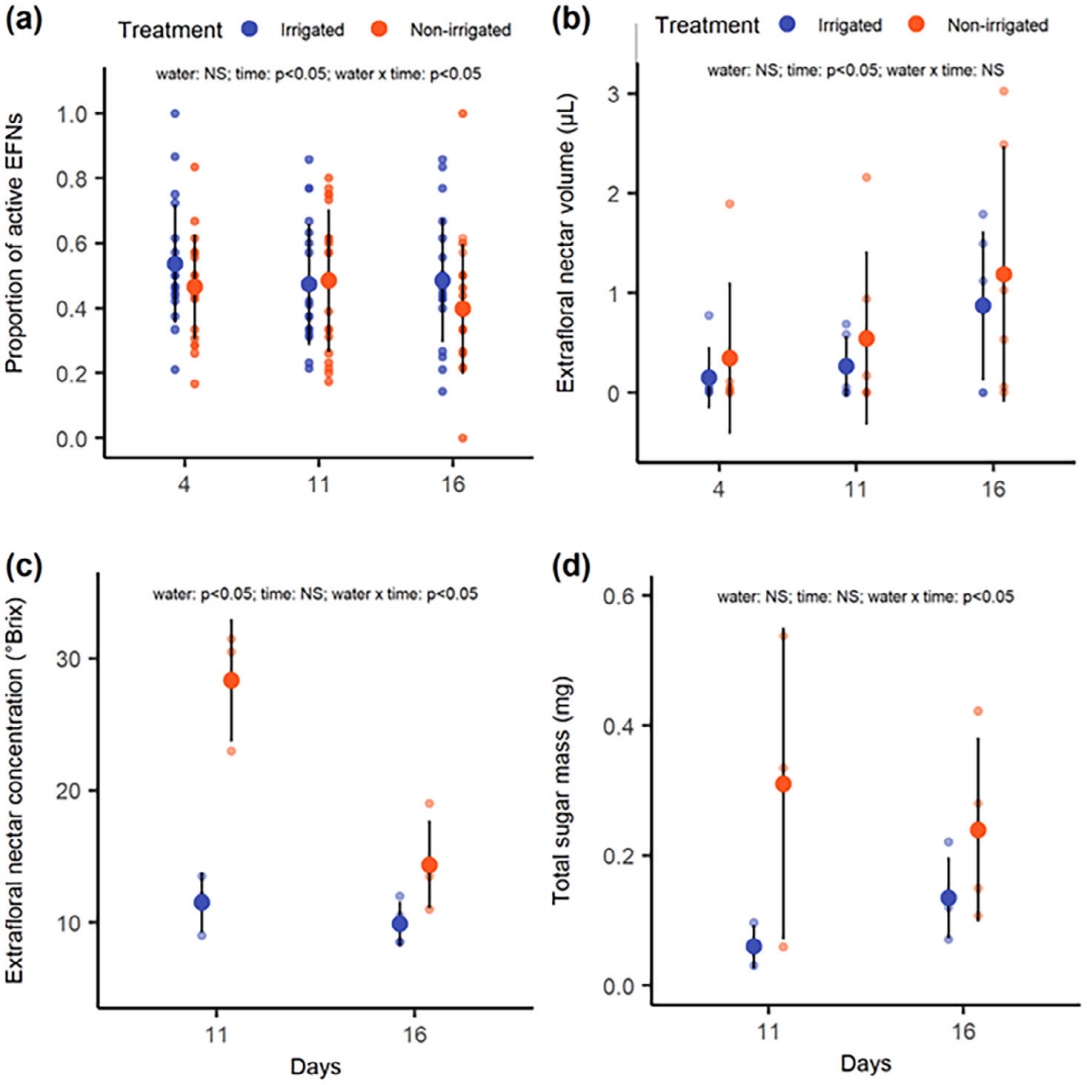
The (a) proportion of active extrafloral nectaries (b) volume, (c) concentration and (d) total sugar mass of the extrafloral nectar secreted by *Chamaecrista nictitans* (Fabaceae) along 21 days under irrigated (blue) and non‐irrigated (orange) treatments. The suspension of irrigation led to a decline in the proportion of active EFNs and the concentration and sugar mass of the extrafloral nectar secreted by the plants. However, the volume of extrafloral nectar increased over the days, regardless of the water availability treatments. The dots represent the mean values, and the bars indicate the standard deviation.

The average proportion of leaves with active EFNs was 0.50 ± 0.19 for irrigated plants and 0.46 ± 0.20 for non‐irrigated plants. However, there was a slight decline in the proportion of active EFNs in non‐irrigated plants over time, while the proportion of leaves with active EFNs remained relatively constant in irrigated plants (GLMM with binomial distribution: χ^2^: 15.6, df = 5, *P* = 0.008). This decline in the proportion of leaves with active EFNs was only detected in the survey conducted on the 16th day of the experiment, with the mean proportion of leaves with active EFNs being 1.13 times lower in the non‐irrigated plants than in the irrigated plants (Fig. 2a).

The average nectar volume between plants at the irrigated and non‐irrigated treatment was 0.42 ± 0.57 μl and 0.69 ± 1.01 μl, respectively. However, the nectar volume was not affected bey water treatment, but independent of water treatment, there was a two‐fold increase in nectar volume over time, from the first to the last day of the survey (GLMM with gamma distribution: χ^2^ = 17.022, df = 5, *P* = 0.004; Fig. 2b). Regarding nectar concentration, the extrafloral nectar secreted by non‐irrigated plants was, on average, 1.6 times more concentrated than the one from irrigated plants (19.2 ± 8.34 and 11.8 ± 3.81, respectively). While the nectar concentration remained relatively constant on irrigated plants, it markedly varied over time in plants from non‐irrigated treatment (LMM with normal distribution: χ^2^ = 29.08, df = 3, *P* < 0.001; Fig. 2c). In the first sample taken 11 days after the onset of the experiment, the concentration of extrafloral nectar secreted by non‐irrigated plants was 2.46 times higher than the one secreted by irrigated plants. This difference gradually declined to a 1.24‐fold higher concentration in the second sample, taken 16 after the irrigation suspension (Fig. 2c). The sugar mass content of the extrafloral nectar followed a similar pattern to the nectar concentration. On average, the sugar mass of the extrafloral nectar secreted by non‐irrigated plants was 2.7 times higher than those secreted by irrigated plants (0.27 ± 0.17 and 0.10 ± 0.10, respectively). However, the magnitude of this difference markedly declined between samples, from 5.18 in the first sample to 2.04 in the second sample (GLMM with gamma distribution: χ^2^ = 8.839, df = 3, *P* = 0.031; Fig. 2d).

### Effect of soil water availability on ant attendance

During the experiment, our plants were visited by 10 ant morphotypes (Supporting Information S1). The most common ant morphotype attending plants in both irrigated and non‐irrigated treatments was *Solenopsis* sp1. and *Camponotus* sp.1, with *Solenopsis* sp.1 more frequent in plants in irrigated and *Camponotus* sp.1 in non‐irrigated treatment (Table S3, Fig. S3). Ants were absent in 62.5% of observations for plants in the irrigated treatment and 70.17% of the observations for plants in the non‐irrigated treatment.

**Fig. 3:**
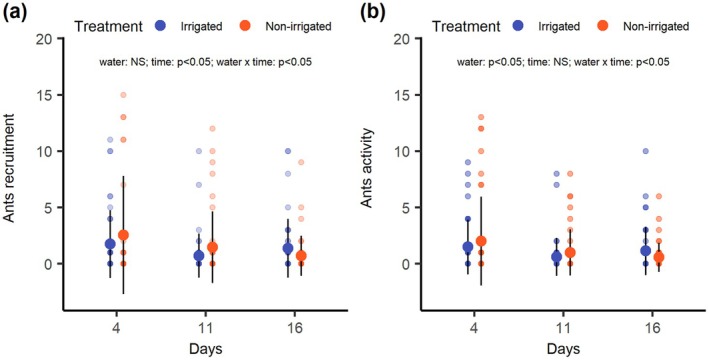
The (a) number and (b) activity of ant workers on individuals of *Chamaecrista nictitans* (Fabaceae) along 21 days under irrigated (blue) and non‐irrigated (orange) treatments. The suspension of irrigation led to increased ant recruitment and activity in *C. nictitans* individuals, followed by a reduction as the irrigation suspension continued. Each point represents an individual *C. nictitans* plant. The dots represent the mean values, and the bars indicate the standard deviation.

Irrigated and non‐irrigated plants had equal chances of being attended by ants along the experiment (GLMM with binomial distribution: χ^2^ = 2.60, df = 3, *P* = 0.46). However, we observed a marked difference in the number of workers attending the plants from each treatment (GLMM with Poisson distribution: χ^2^ = 4.81, df = 6, *P* = 0.02). Initially, non‐irrigated plants attracted on average 2.5 times more ant workers than irrigated plants. However, this pattern was inverted on the sixteenth day of the experiment, when irrigated plants started to attract more ant workers than non‐irrigated plants (Fig. 3a). Similarly, ants showed higher activity initially on the apical region of non‐irrigated plants, but this pattern was inverted on the 16th day of the experiment, with higher ant activity observed in plants from irrigated treatment (GLMM with negative binomial distribution: χ^2^ = 8.2606, df = 3, *P* = 0.040; Fig. 3b).

## DISCUSSION

Our results showed that while soil moisture declined rapidly in the first 5 days of water scarcity, the leaf's water potential of *C. nictitans* declined only after 16 days of irrigation suspension. This pattern indicates that plants can maintain their hydric status for a certain period after the soil drought begins. As expected, we observed a clear relationship between the decline in water availability and the patterns of extrafloral nectar secretion by *C. nictitans* and, interestingly, this pattern strongly matched the temporal variation in the leaf's water status during our experiment. On the drought onset, while the plants maintained their hydric status, the decline in soil moisture led to an increase in the number of active EFNs in the plants and the concentration and sugar mass of the extrafloral nectar secreted. However, as the drought persisted, interfering with the plant's water potential, plants exhibited less active EFNs, secreting a low‐quality extrafloral nectar in comparison with plants continuously irrigated. Also as expected, the patterns of ant attendance to plants varied predictably following the patterns of extrafloral nectar secretion along the drought period. Although drought intensity did not affect the probability of ant attendance to plants, the irrigation suspension initially increased the number and activity of ant workers on plants, being followed by a marked decline as water scarcity persisted. Altogether, our results shed new light on the physiological processes regulating the extrafloral nectar secretion by plants and indirectly mediating the ant attendance to EFNs along temporal and spatial environmental water gradients. These processes will be discussed in more detail below.

The three extrafloral nectar descriptors (volume, concentration and total sugar mass) measured in the *C. nictitans* plants were not equally affected by progressive water scarcity. While the suspension of irrigation had no effect on the volume of extrafloral nectar secreted over time, it significantly modified its concentration and sugar content. Regarding the concentration and sugar content, its variation throughout our experiment aligns with the expected variation in NSC availability along drought periods. When soil moisture begins to decrease, the roots are the first organ to detect it, immediately triggering hormonal responses to deal with water restriction (*e.g*., increased abscisic acid secretion) (Chaves *et al*. 2002). The initial plant response to it is a decline in the plant's above‐ground growth and posterior modifications in the patterns of the stomatal aperture (*i.e*., they remain closed for longer) (Chaves *et al*. 2002). Our results indicate then that this initial surplus of NSC increases the quality of plant rewards to ant bodyguards, favouring this protective mutualistic interaction. It is important to note that we observed no signs of herbivory in the plant of both treatments, meaning that extrafloral nectar induction by herbivory (see Heil 2011) did not influence the patterns of extrafloral nectar secretion described here for both treatments.

Although subsequent modifications in stomatal aperture as drought lasts interfere with the plant's gas exchange (Mundim & Pringle 2018), plants are still able to absorb CO_2_ and transport NSC while the leaf's water potential is relatively stable, making them less susceptible to carbon starvation (Santos *et al.* 2021; Sharma *et al*. 2019). This scenario is expected to change when drought becomes more severe and compromises the leaf's water potential. Leaf water status is a key factor affecting plant photosynthetic rates and NSC assimilation and transport within the plants (Johnson & Mcculloh 2009; Santos *et al*. 2009; McDowell & Sevanto 2010): the lower the leaf's water potential, the lower the NSC assimilation via photosynthesis (Tezara *et al*. 1999). This is likely the reason for the marked decline in the concentration and sugar content of the extrafloral nectar secreted by *C. nictitans* after the 16th day of the experiment, when the leaf's water potential also began to decline. Although indirect, these results are the first empirical evidence that the energetic cost of the extrafloral nectar intensifies not at the drought beginning, but after a certain period of drought when photosynthesis begins to be compromised, and the NSC content declines in the plant tissues.

Regarding nectar volume, we observed that both irrigated and non‐irrigated plants secreted a higher volume of nectar over time. Such an increase is likely due to the production of new leaves with new EFNs by the plants along the experiment. In our experiment, some plants of both treatments lost their oldest leaves, but new leaves expanded (personal observation), replacing the no longer nectar‐producing leaves with new nectar‐producing leaves. These falling leaves, however, were not related to a decline in water availability for the plants, but mostly old senescent leaves naturally falling from *C. nictitans* along its life cycle. Even so, it is interesting that a decline in water availability did not interfere with the magnitude of nectar volume increasing since extrafloral nectar is primarily composed of water. It suggests that *C. nictitans* can still transport water through the xylem to EFNs even when water potential is slightly reduced. In fact, the maintenance of water transportation through the xylem at moderate to low water potential is a relatively common phenomenon among annual plant species (*e.g*., Lauteri *et al*. 1997; Kooyers 2015; Kulkarni *et al*. 2017). Different from other types of abiotic stresses, plant drought stress is not abrupt, developing slowly as water scarcity lasts, with plants exhibiting subsequent responses to it (Larcher 2003). Therefore, it is likely that drought in our experiment was enough to modify patterns of plant carbohydrate allocation to extrafloral nectar secretion, but not water. After the 21st day of the experiment, non‐irrigated plants were dead due to drought, with no regrowth signal, meaning that *C. nictitans* can likely maintain the volume of extrafloral nectar secreted along the drought period until the plants start to shed and lose the EFNs.

Regarding the drought effects on the patterns of ant visitation, we observed contrasting effects of water scarcity on the probability of ant attendance, recruitment, and activity on the plants. This apparent divergence provides an opportunity to elucidate the indirect effects of drought on plant attractiveness to ants and the associated mechanisms. While the relative quality of extrafloral nectar determines plant attractiveness to ants (Ness *et al*. 2006; Fagundes *et al*. 2017; Flores‐Flores *et al*. 2018; Souza *et al*. 2022), ant attendance to plants is also influenced by extrinsic factors, such as the spatial distribution of plants, ant nests (Dáttilo *et al*. 2013; da Silva *et al*. 2019) and the availability of alternative carbohydrate food sources for ants in the habitat (Passos & Leal 2019). Despite that, plants are more likely to be defended against herbivores when more workers are patrolling them (Rosumek *et al*. 2009; Xu & Chen 2010; Yamawo *et al*. 2012). Similarly, increasing in the relative quality of extrafloral nectar favours the plant interaction with dominant ant species, which are more effective bodyguards due to their numerical advantage and behavioural capacity to monopolize and defend resources (Leal et al. 2022, but see Melati & Leal 2018). Altogether, it suggests that the progressive decline in soil water availability plays a minor role in the chances of ant occurrence on the plants but can strongly affect their identity and the way these ants forage on plants. Drought had a similar effect on *Cylindropuntia imbricata* (Cactaceae), in which irrigation suspension led to a decline in ant abundance on the plants and a marked change in the ant assemblage visiting their EFNs (Ávila‐Argáez *et al*. 2019). Our results indicate then that plants will be defended more efficiently, either by interacting more with dominant ants or attracting more ants in general, when water availability starts to decline in the soil, triggering the plant's investment in extrafloral nectar secretion. This efficiency is expected to decline when drought compromises the plant's water potential, leading to changes in the patterns of extrafloral nectar secretion.

Although our results came from plants under controlled conditions, they bring important implications to our comprehension about the temporal dynamic of ant–EFN interaction in natural environments. In our experiment, the plants grew in a restricted environment, relying only on the water content of the soil available in each pot. This allowed us to detect plant physiological responses to changes in soil water conditions over a short period of time (21 days). In nature, plant's roots cover a larger soil area than those available in our experiments. Also, the soil moisture declines slower in nature than in our pots. Consequently, changes in the patterns of extrafloral nectar secretion in response to decline in soil water availability under natural conditions should occur over larger temporal scales than we observed in our experiment, along dry seasons in seasonal environments, for instance. Although this effect also depends on the plant phenology (Nogueira *et al*. 2020), we should expect an increase in the sugar content in extrafloral nectar at the beginning of the dry season if EFNs are active. This increased sugar secretion can momentarily enhance plant defence against herbivory by attracting more protective ants. However, as the dry season progresses, plant investment in sugar allocation to extrafloral nectar should decline, reducing the plant defence by ant bodyguards throughout the rest of the dry season. The potential effects of climatic changes on this mutualistic ant–plant interaction represent another significant implication of our results. In the coming years, drought periods are expected to become more frequent, longer and increasingly unpredictable (Karl & Trenberth 2003; Archer & Rahmstorf 2010). Based on our results, this future scenario will likely compromise the plant's ability to secrete extrafloral nectar, threatening the maintenance of this indirect anti‐herbivory defence in plants with EFNs. Although our generalization is based on one model species, many plants bearing EFNs are small shrubs in open habitats, like *C. nictitans*. These species typically possess smaller and shallower root systems than long‐lived large shrub or tree species (*e.g*., Bliss *et al*. 2002), making them less tolerant to severe droughts. If future climatic scenarios unfold as projected, it is expected to severely compromise the effectiveness of indirect anti‐herbivory defence in those plant species, potentially leading to the collapse of this ant‐plant mutualism in several ecosystems worldwide.

## AUTHOR CONTRIBUTIONS

BM, LCL and AN designed the study. BM and CS performed experiment setup with the legume species. BM data collection was carried out. BM performed analyses. LCL and AN provided funds. BM wrote the first draft of the manuscript, and all authors commented on previous versions. All authors helped with data interpretation, writing article, and approving the final manuscript.

## Supporting information


**Table S1.** Values of water curve retention for a mixed soil composed of 70% sand and 30% garden soil rich in organic matter and macronutrients (Terra Vegetal ABC GARDEN), simulating the soil natural conditions experienced by *Chamaescrista nictitans*. The analysis was estimated by the Laboratório de Física do Solo, Instituto Agronômico (soil physics laboratory, Agronomic Institute) – Universidade de Campinas (UNICAMP).
**Table S2.** Polynomial regression models evaluating the variation in soil moisture and plant water potential along the experiment. The models with the best fit, determined by Akaike's information criteria (AIC), are highlighted. The selected models have the smallest order and AIC, indicating the best fit.
**Table S3.** Relative frequency of *Chamaescrista nictitans* (Fabaceae) attendance by different ant morphotypes under different water regimes (irrigated and non‐irrigated). The two most frequent ant morphotypes attending plants in both treatments are highlighted in bold.
**Fig. S1.** Five‐month‐old *Chamaecrista nictitans* (Fabaceae) individuals used in the experiment. (a) Plant growing in the experimental pots before the application of the irrigation suspension treatment; (b) extrafloral nectary secreting nectar; (c) *Camponotus* sp. ant visiting a *C. nictitans* individual during the experiment; and (d) non‐irrigated treatment pots covered with a plastic lid to prevent rainwater ingress.
**Fig. S2.** Schematic flowchart representing the experimental design. (A) We cultivated 48 *Chamaecrista nictitans* (Fabaceae) seedlings from seeds in a greenhouse from December to June 2020. In July 2020, all plants were transferred to our study site and allocated into two different parallel experiments: (B) one for monitoring soil and leaf water status and other for evaluating EFN activity and ant visitation. These two experiments lasted 21 days.
**Fig. S3.** Interaction frequency of ants visiting individuals of *Chamaecrista nictitans* under (a) irrigated, (b) non‐irrigated and (c) both irrigated and non‐irrigated treatments. Dot refers to ant morphotypes, lines connecting points and ant morphotypes names relate the ant morphotype representing that dot. Blue and orange are ants visiting plants under irrigated treatment and non‐irrigated treatment respectively.
**Fig. S4.** Extrafloral nectar volume and total sugar mass of *Chamaecrista nictitans* individuals controlled by the size of plants. As a proxy of size, we used the number of leaves and the number of leaves with active EFNs. In (a, b) the volume controlled by the number of leaves and leaves with active EFNs. In (c, d) the total sugar mass controlled by the number of leaves and leaves with active EFNs. Blue and orange points indicate plants at irrigated and non‐irrigated treatments, respectively. The bigger dots represent the mean and bars represent standard deviation.
