# Supplementary material for: Water availability shapes temporal patterns of extrafloral nectar secretion and ant visitation to a Neotropical legume
Source: Plant Biol (Stuttg). 2026 Jan 5;28(3):913–23. doi: 10.1111/plb.70164 (PMC13089596; doi:10.1111/plb.70164)
Supplement: Supplementary file 1 — Table S1. Values of water curve retention for a mixed soil composed of 70% sand and 30% garden soil rich in organic matter and macronutrients (Terra Vegetal ABC GARDEN), simulating the soil natural conditions experienced by Chamaescrista nictitans. The analysis was estimated by the Laboratório de Física do Solo, Instituto Agronômico (soil physics laboratory, Agronomic Institute) – Universidade de Campinas (UNICAMP). Table S2. Polynomial regression models evaluating the variation in soil moisture and plant water potential along the experiment. The models with the best fit, determined by Akaike's information criteria (AIC), are highlighted. The selected models have the smallest order and AIC, indicating the best fit. Table S3. Relative frequency of Chamaescrista nictitans (Fabaceae) attendance by different ant morphotypes under different water regimes (irrigated and non‐irrigated). The two most frequent ant morphotypes attending plants in both treatments are highlighted in bold. Fig. S1. Five‐month‐old Chamaecrista nictitans (Fabaceae) individuals used in the experiment. (a) Plant growing in the experimental pots before the application of the irrigation suspension treatment; (b) extrafloral nectary secreting nectar; (c) Camponotus sp. ant visiting a C. nictitans individual during the experiment; and (d) non‐irrigated treatment pots covered with a plastic lid to prevent rainwater ingress. Fig. S2. Schematic flowchart representing the experimental design. (A) We cultivated 48 Chamaecrista nictitans (Fabaceae) seedlings from seeds in a greenhouse from December to June 2020. In July 2020, all plants were transferred to our study site and allocated into two different parallel experiments: (B) one for monitoring soil and leaf water status and other for evaluating EFN activity and ant visitation. These two experiments lasted 21 days. Fig. S3. Interaction frequency of ants visiting individuals of Chamaecrista nictitans under (a) irrigated, (b) non‐irrigated and (c) both irrig [file PLB-28-913-s001.docx]

**Supplementary Information**

**Table S1.** Values of water curve retention for a mixed soil composed of f 70% sand and 30% garden soil rich in organic matter and macronutrients (Terra Vegetal ABC GARDEN), simulating the soil natural conditions experienced by *Chamaescrista nictitans*. The analysis was estimated by the Laboratório de Física do Solo, Instituto Agronômico (soil physics laboratory, Agronomic Institute) – Universidade de Campinas (UNICAMP).

| **Soil Moisture** | | | | **Density of the soil by the beaker** |
| --- | --- | --- | --- | --- |
| **Saturated Soil** | **1 kPa** | **5 kPa** | **10 kPa** |  |
| ^__________________________________________________________________________^  m^3^ m^-3^  ^__________________________________________________________________________^ | | | | Mg m^-3^ |
| 0,4587 | 0,3091 | 0,1862 | 0,1394 | 1,39 |

**Table S2.** Polynomial regression models evaluating the variation in soil moisture and plant water potential along the experiment. The models with the best fit, determined by Akaike’s Information Criteria (AIC), are highlighted. The selected models have the smallest order and AIC, indicating the best fit.

| **Order** | **AICc** | **dAICc** | **weight** | **df** | **p** | **r²** |
| --- | --- | --- | --- | --- | --- | --- |
|  | |  |  |  |  |  |
| *- Soil moisture* | |  |  |  |  |  |
| $\mathbf{5}^{\boldsymbol{th}}$ | **222.0** | **0.0** | **0.5** | **13** | **<0.001** | **0.96** |
| $6^{th}$ | 222.0 | 0.0 | 0.5 | 13 | <0.001 | 0.96 |
| $4^{th}$ | 249.1 | 27.1 | <0.001 | 11 | <0.001 | 0.93 |
| $2^{nd}$ | 257.6 | 35.5 | <0.001 | 5 | <0.001 | .0.91 |
| $3^{th}$ | 258.2 | 36.2 | <0.001 | 9 | <0.001 | 0.88 |
| $1^{st}$ | 266.6 | 44.5 | <0.001 | 5 | <0.001 | 0.86 |
|  |  |  |  |  |  |  |
| *- Water potential* | |  |  |  |  |  |
| $\boldsymbol{4}^{\boldsymbol{th}}$ | **61.1** | **0.0** | **0.907** | **11** | **<0.001** | **0.98** |
| $5^{th}$ | 67.1 | 6.0 | 0.046 | 13 | <0.001 | 0.98 |
| $6^{th}$ | 67.1 | 6.0 | 0.046 | 13 | <0.001 | 0.98 |
| $3^{th}$ | 89.1 | 28.0 | <0.001 | 9 | <0.001 | 0.96 |
| $1^{st}$ | 194.7 | 133.6 | <0.001 | 5 | <0.001 | 0.58 |
| $2^{nd}$ | 227.7 | 166.6 | <0.001 | 5 | 0.21 | 0.17 |

**Table S3.** Relative frequency of *Chamaescrista nictitans* (Fabaceae) attendance by different ant morphotypes under different water regimes (irrigated and non-irrigated). The two most frequent ant morphotypes attending the plants in both treatments are highlighted in bold.

|  |  |  |  |
| --- | --- | --- | --- |
| **Species** | **total** | **irrigated** | **non-irrigated** |
| ***Camponotus sp****.* | **0.24** | **0.17** | **0.35** |
| *Crematogaster sp.* | 0.02 | 0.04 | 0.00 |
| *Dorymyrmex sp1.* | 0.04 | 0.06 | 0.00 |
| *Dorymyrmex sp2.* | 0.12 | 0.15 | 0.09 |
| *Linepthema sp.* | 0.02 | 0.00 | 0.06 |
| *Nylanderia sp.* | 0.04 | 0.06 | 0.00 |
| *Pheidole sp.* | 0.16 | 0.13 | 0.21 |
| *Pseudomyrmex sp.* | 0.07 | 0.06 | 0.06 |
| ***Solenopsis sp.*** | **0,26** | **0,28** | **0,24** |
| *Brachymyrmex sp.* | 0,02 | 0,04 | 0,00 |

**Fig. S1**


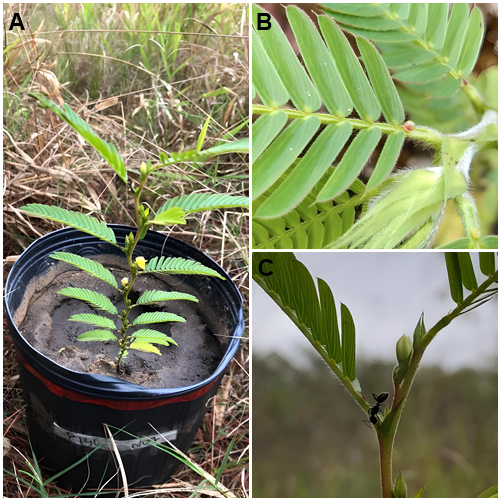

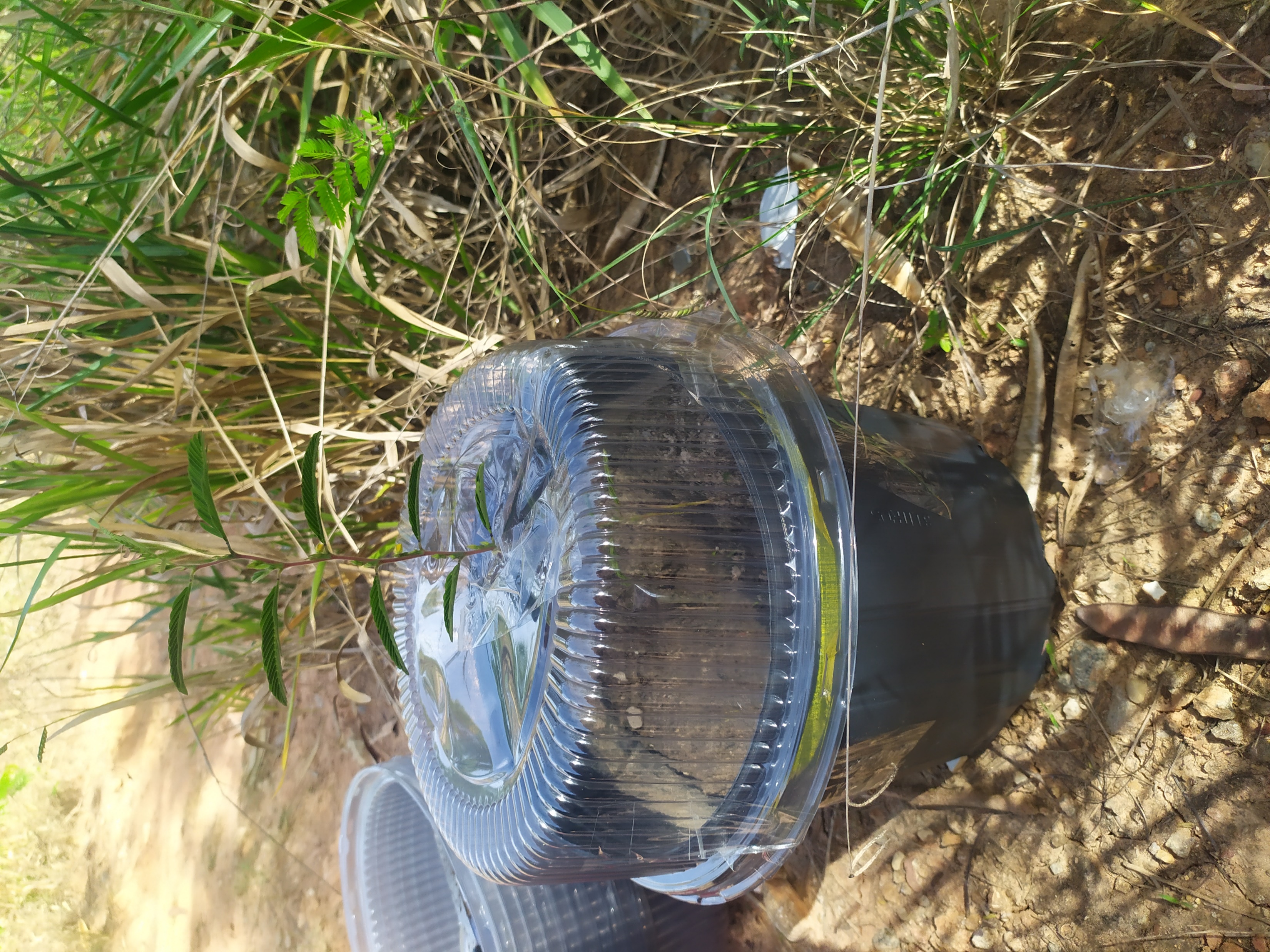

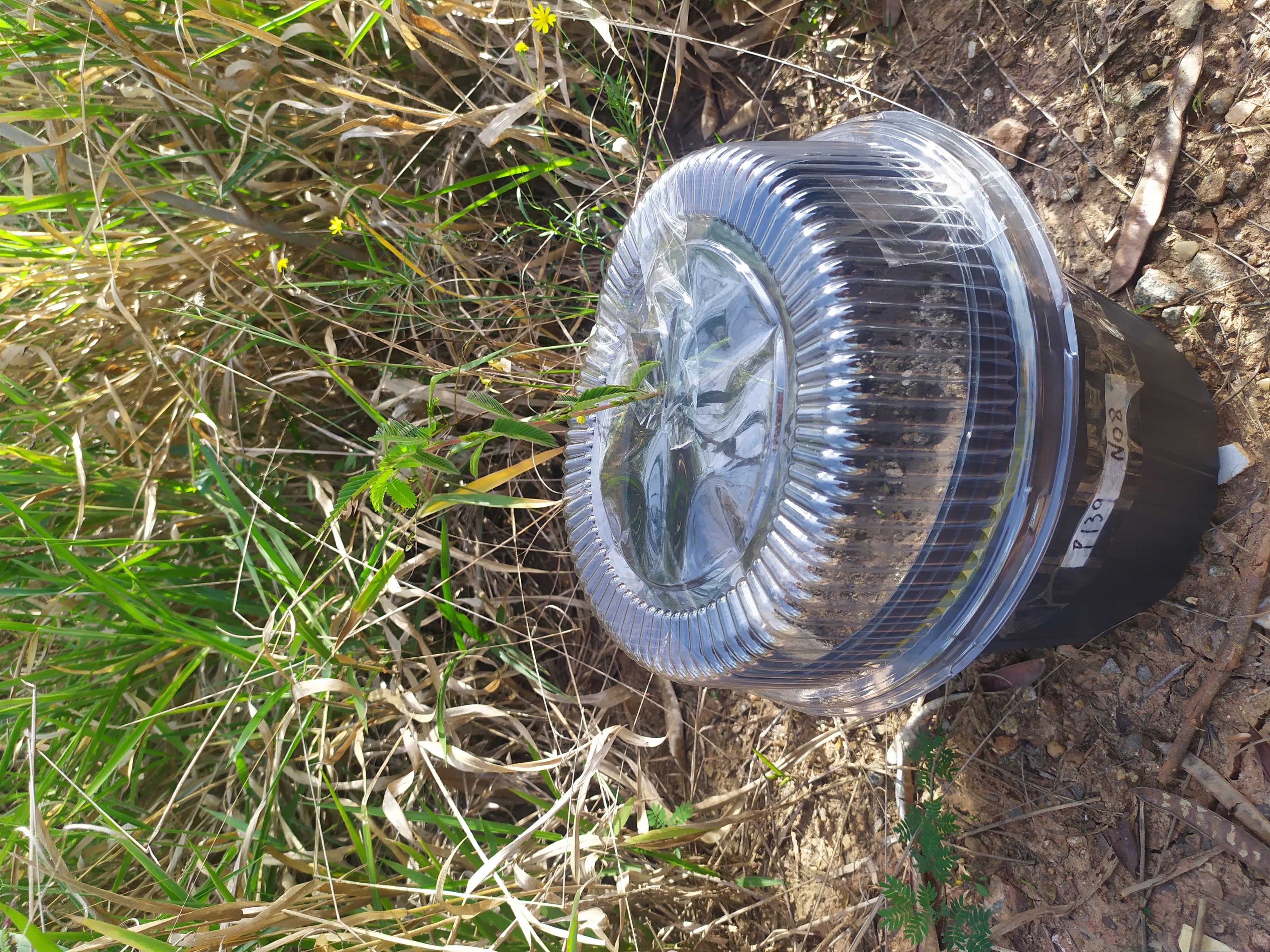

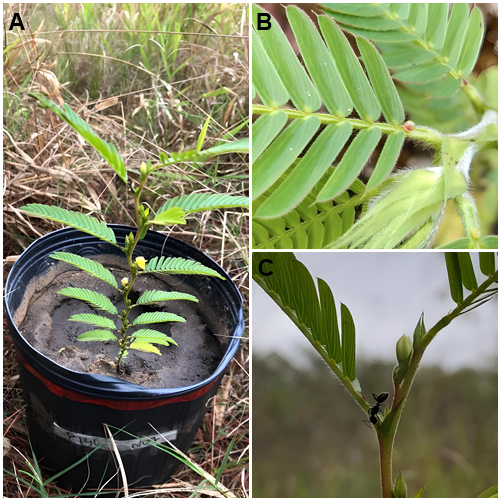

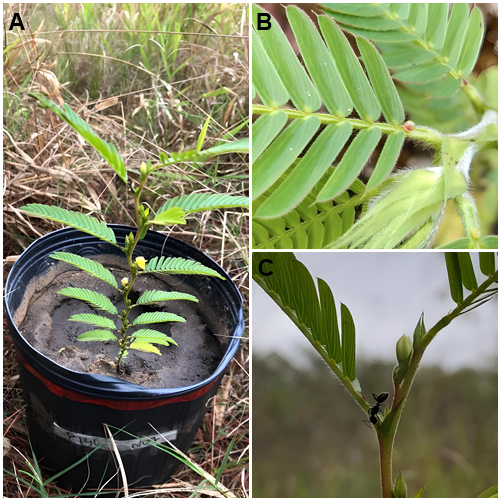


**D**

**E**

**D**

**E**

**Fig. S1.** Five-month-old *Chamaecrista nictitans* (Fabaceae) individuals used in the experiment. (a) Plant growing in the experimental pots before the application of the irrigation suspension treatment; (b) Extrafloral nectary secreting nectar; (c) *Camponotus* sp. ant visiting a *C. nictitans* individual during the experiment; and (d) Non-irrigated treatment pots covered with a plastic lid to prevent rainwater ingress*.*

**Fig. S2**

**
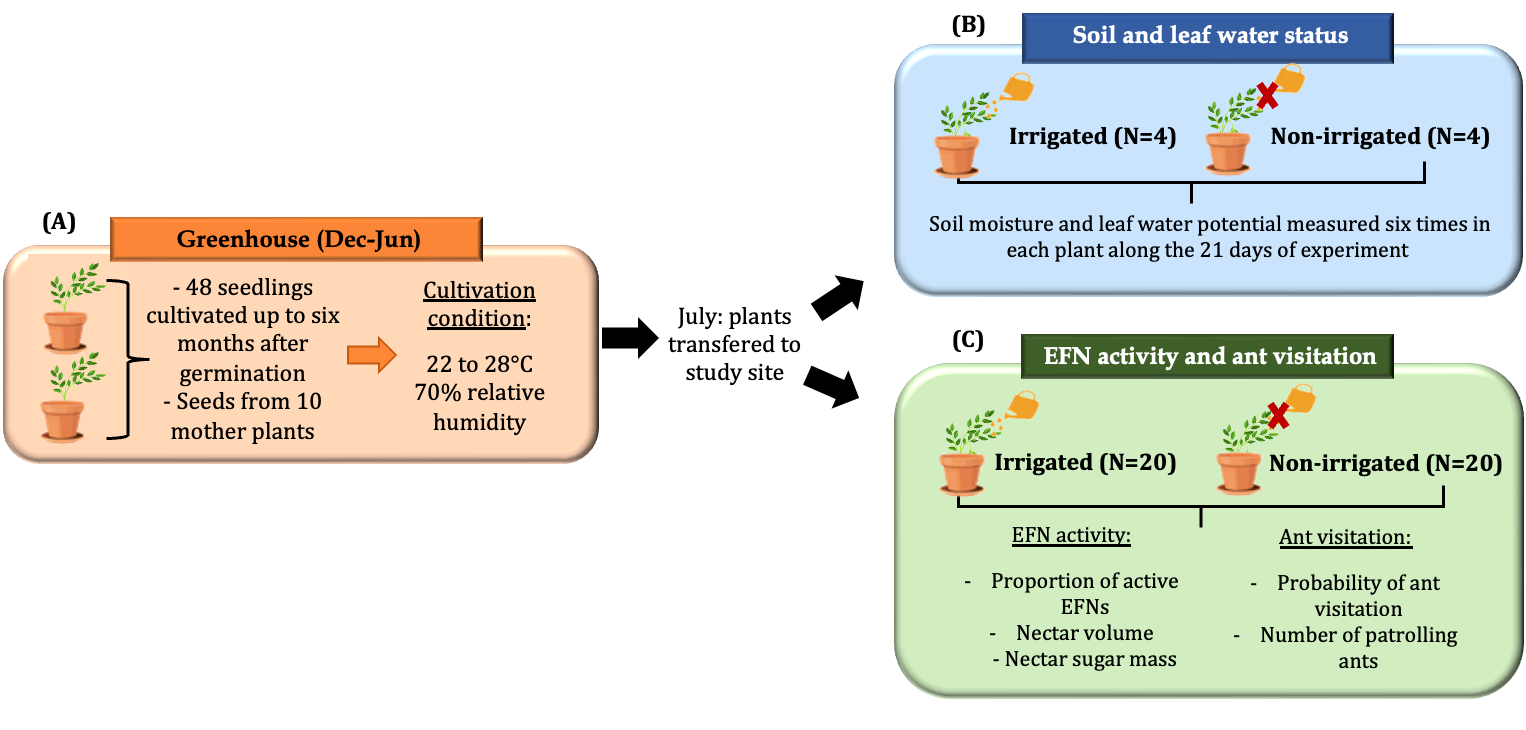
**

**Figure S2.** Schematic flowchart representing the experimental design. (A) We cultivated 48 *Chamaecrista nictitans* (Fabaceae) seedlings from seeds in a greenhouse from December to June 2020. In July 2020, all plants were transferred to our study site and allocated into two different parallel experiments: (B) one for monitoring soil and leaf water status and (ii) other for evaluating EFN activity and ant visitation. These two experiments lasted 21 days.

**Fig. S3**


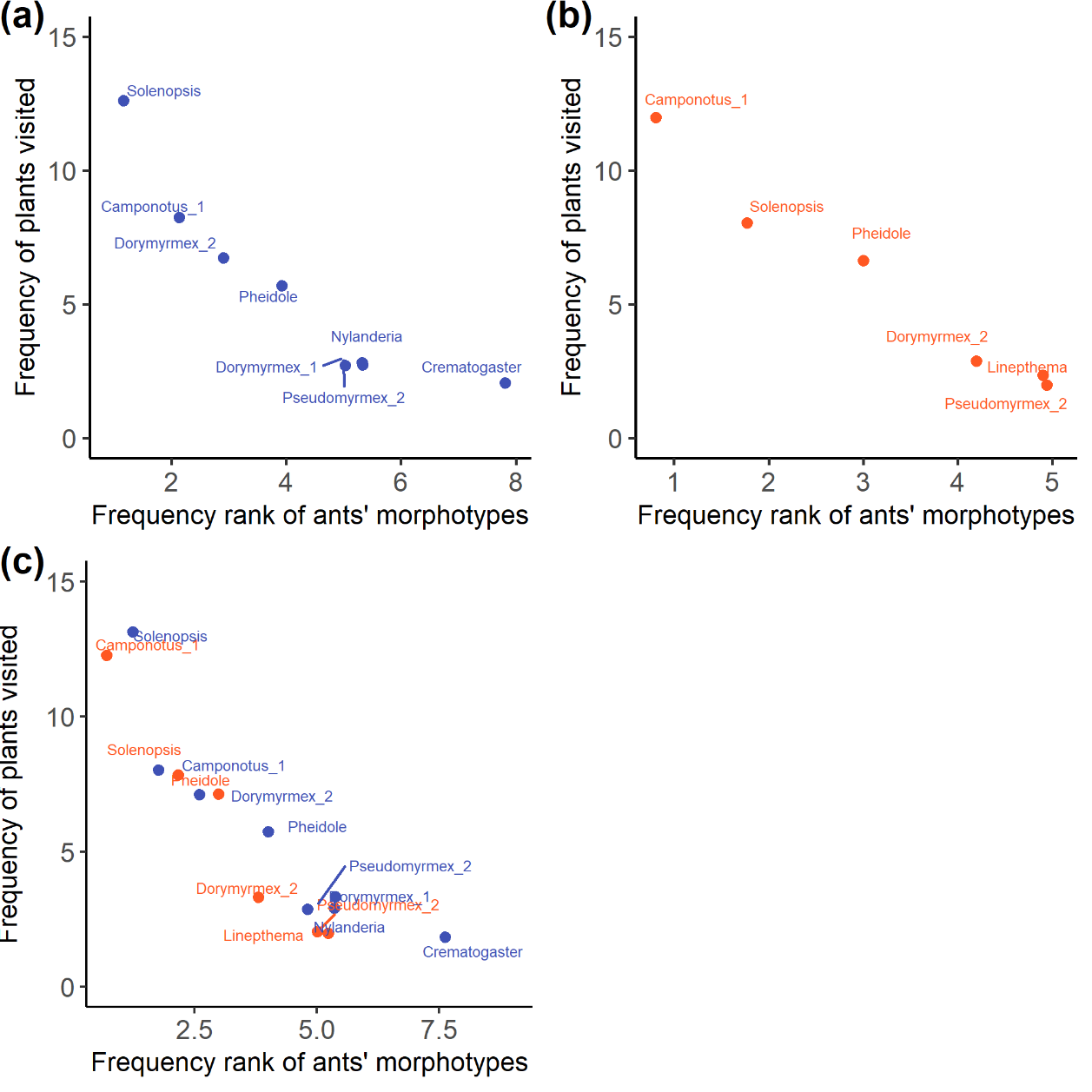


**Figure S3.** Interaction frequency of ants visiting individuals of *Chamaecrista nictitans* under (a) irrigated, (b) non-irrigated and (c) both irrigated and non-irrigated treatments. Dot refers to ant morphotypes, lines connecting points and ant morphotypes names relate the ant morphotype representing that dot. Blue and orange are ants visiting plants under irrigated treatment and non-irrigated treatment respectively.

**Fig. S4**


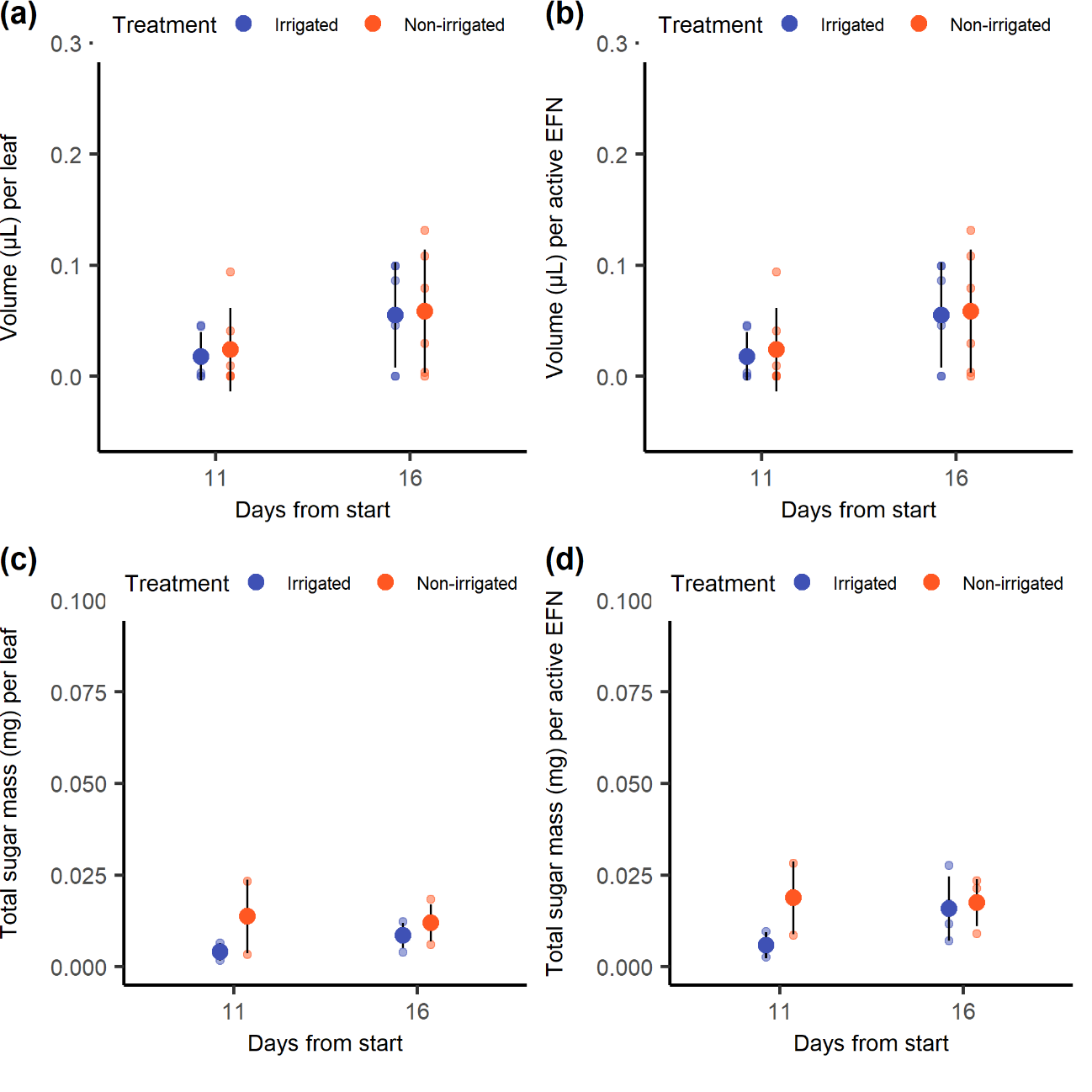


**Figure S4.** Extrafloral nectar volume and total sugar mass of *Chamaecrista nictitans* individuals controlled by the size of plants. As a proxy of size, we used the number of leaves and the number of leaves with active EFNs. In (a) and (b) the volume controlled by the number of leaves and leaves with active EFNs. In (c) and (d) the total sugar mass controlled by the number of leaves and leaves with active EFNs. Blue and orange points indicate plants at irrigated and non-irrigated treatments, respectively. The bigger dots represent the mean and bars represent standard deviation.
